# Pediatric Nasopharyngeal Carcinoma as Seen on 18F-FDG PET/CT

**DOI:** 10.3389/fonc.2019.00110

**Published:** 2019-03-06

**Authors:** Razi Muzaffar, Francesca Vacca, Huazhang Guo, Rishi Mhapsekar, Medhat M. Osman

**Affiliations:** ^1^Division of Nuclear Medicine, Department of Radiology, Saint Louis University, Saint Louis, MO, United States; ^2^Department of Pathology, Saint Louis University, Saint Louis, MO, United States; ^3^Department of Radiology, Saint Louis University, Saint Louis, MO, United States

**Keywords:** pediatrics, nasopharyngeal carcinoma, PET/CT, FDG, Epstein-Barr virus

## Abstract

Pediatric nasopharyngeal carcinoma is a rare malignancy strongly associated with Epstein-Barr virus infection. Patients typically present with non-specific symptoms of epistaxis or serous otitis from eustachian tube obstruction and therefore diagnosis is often delayed. We present a case of a previously healthy 17 year old female who initially complained of migraines which was resistant to oral medication. Symptoms progressed and she saw a dental surgeon for concern of a dental infection and was prescribed antibiotics with no relief. Her symptoms continued to progress until an otolaryngologist visualized a large mass along the floor of the left nasal cavity. Subsequent imaging showed a large mass in the posterior left nasal cavity and biopsy was consistent with nasopharyngeal carcinoma.

## Case Presentation

**Figure 1:**
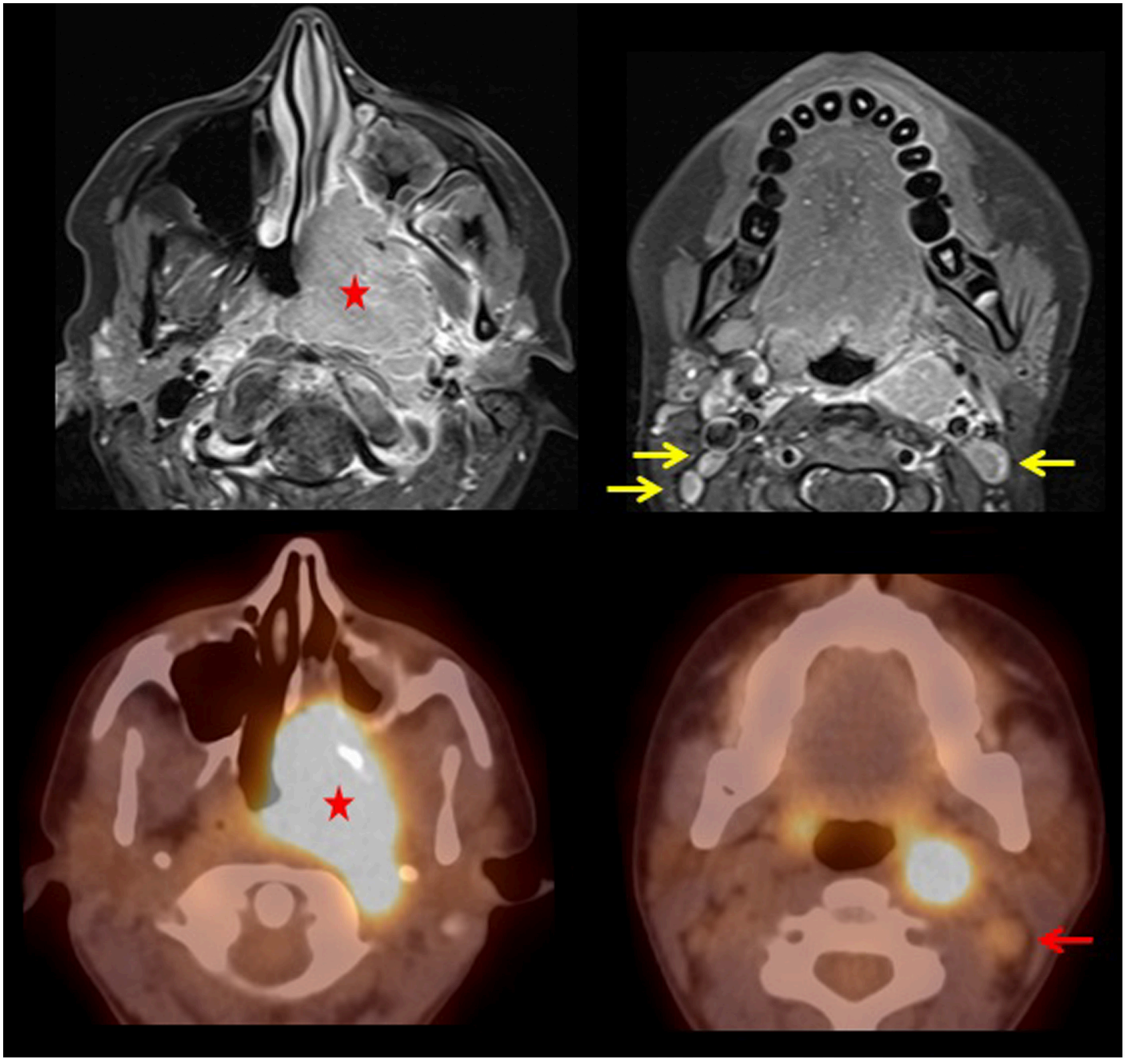
T1 post-contrast fat saturated MRI images (top row) demonstrates diffuse enhancement of the large aggressive mass in the posterior left nasal cavity and nasopharynx (star) invading the left maxillary sinus, nasal septum, left pterygopalatine fossa, left masticator and pharyngeal spaces, and left longus colli muscle causing narrowing of the nasal airway and nasopharynx along with cervical lymphadenopathy (yellow arrows). Fused PET/CT images (bottom row) demonstrate a large FDG avid mass in the left nasal cavity with extension to the maxillary sinus and nasopharynx. There is also an enlarged FDG avid left level 2 cervical lymph node (red arrow).

**Figure 2:**
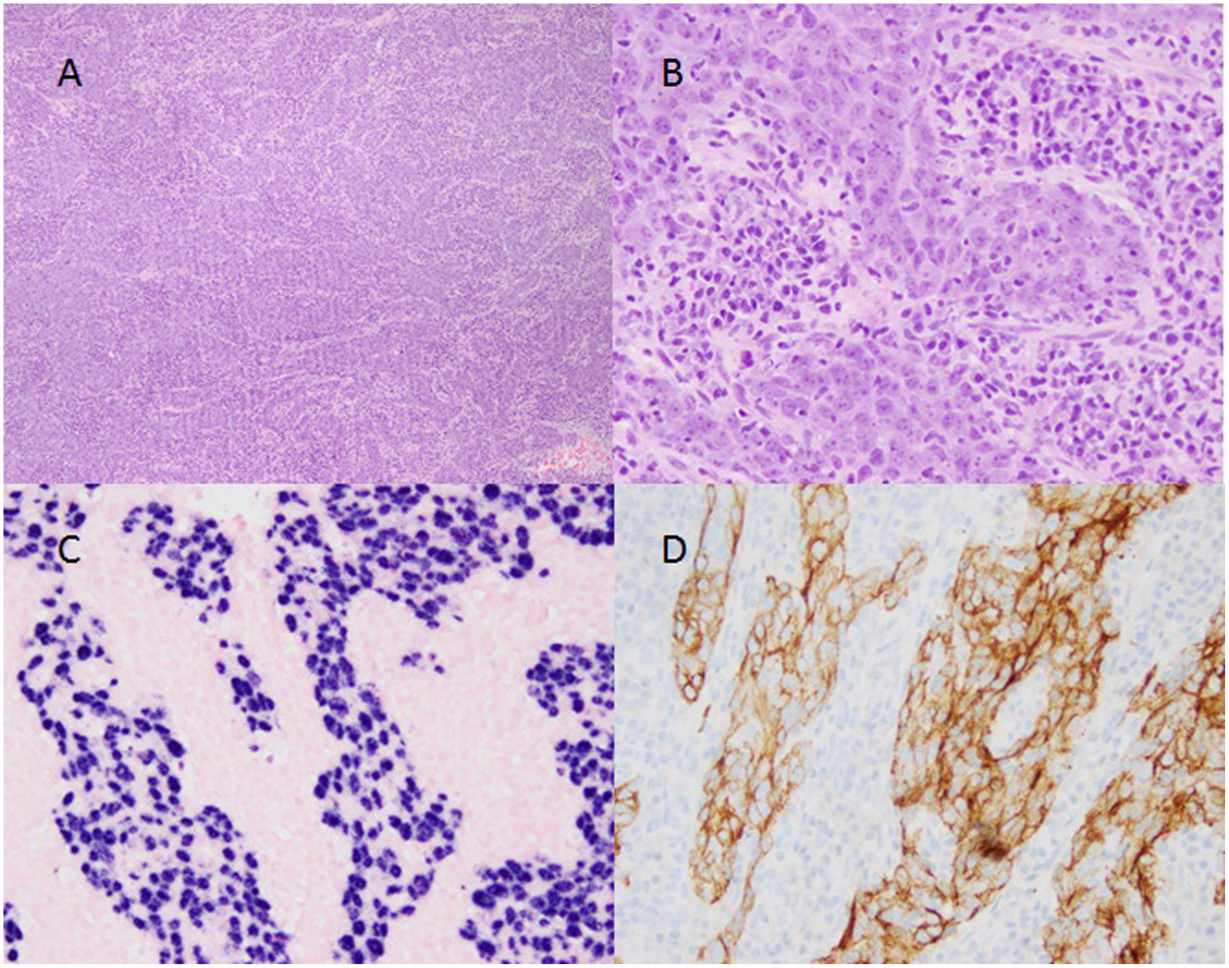
Biopsy of nasopharyngeal carcinoma. (**A**, 4x magnification) Large epithelioid cells divided by sheets of small dark lympho-plasmacytic cells demonstrating the biphasic nature of the tumor. (**B**, 20x magnification) Large pleomorphic nuclei with open chromatin and prominent nucleoli within the epithelioid component and both plasmacytic and lymphocytic differentiation within the lymphoid component. (**C**, 20x magnification) Positive staining (violet) within the nuclei of the large epithelioid cells and negative staining (pink) of the lympho-plasmacytic cells indicating the presence of Epstein-Barr Virus RNA expression in the epithelioid component. (**D**, 20x magnification) positive CK-5/6 immunohistochemical staining (brown) in a membranous distribution on the epithelioid cells with negative staining (blue) on the lympho-plasmacytic cells.

We present a previously healthy 17 year old female who initially complained of migraines resistant to ibuprofen and imitrex. Symptoms progressed over the next 3–4 months to nasal congestion and she became unable to breathe through the left nostril. She saw a dental surgeon for trismus due to concern of a dental infection and was prescribed antibiotics (Amoxicillin) with no relief. Her symptoms progressed over another 3 months to unexplained weight loss, headaches, loss of hearing, frequent bloody nose, tooth pain, and hoarseness. Approximately 9 months after her initial symptoms, she eventually was seen by ENT and a rigid nasal endoscopy was performed which demonstrated a polypoid mass along the floor of the left nasal cavity. MRI with and without gadolinium contrast performed on a 3-T Siemens Skyra showed a large mass in the posterior left nasal cavity and nasopharynx invading the left maxillary sinus, nasal septum, left pterygopalatine fossa, left masticator and pharyngeal spaces and left longus colli muscle causing narrowing of the nasal airway and nasopharynx as well as a unilateral mastoid effusion (Figure 1, top row). The top differential considerations included adenoidal benign lymphoid hyperplasia, nasopharyngeal non-Hodgkin and Hodgkin lymphoma, juvenile angiofibroma, nasopharyngeal rhabdomyosarcoma, and nasopharyngeal carcinoma. Subsequent biopsy of the mass was consistent with non-keratinizing nasopharyngeal carcinoma (Figure 2). She then presented for an ^18^F-FDG PET/CT study performed on a Philips Gemini Time of Flight system imaged from the top of the skull to the feet (FDG dose 6.8 mCi). The scan demonstrated an intense FDG avid mass in the left nasopharynx and nasal cavity extending to the left maxillary sinus (SUV max 10.8) and a metastatic left cervical lymph node (SUV max 6.9) with no distant metastasis (Figure 1, bottom row). EBV panel and PCR were both strongly positive (see Discussion section). According to the TNM classification, the tumor was classified as T3 (tumor has grown into the sinuses and/or bones nearby), N2 (spread to nearby lymph nodes no larger than 6 cm), and M0 (no distant metastasis), stratifying her to stage III disease. She has recently completed neoadjuvant chemotherapy (cisplatin and 5-fluorouracil) and radiation therapy and currently under strict follow-up. This retrospective study was approved by the Institutional Review Board and the informed consent was obtained.

## Discussion

Nasopharyngeal carcinoma (NPC) is a mucosal tumor of the lateral pharyngeal recess and is a relatively rare disease in the US with an annual incidence of 0.5–2.0 cases per 100,000. Peak incidence is 40–60 years with males being at a higher risk. NPC is a rare malignancy in children accounting for <1% of all pediatric neoplasms. They typically present at a later stage in children due to the non-specific symptoms of epistaxis, headaches, nasal congestion, and conductive hearing loss secondary to middle ear obstruction. There is a strong association of NPC with Epstein-Barr Virus (EBV) infection where it is found in 90–100% of cases (1–3). In our case, lab values were positive for EBV infection: EBV qPCR was 2,900 IU/mL (normal not detected), EBV Ab VCA IgM <36.0 U/mL (positive >43.9), EBV Early Antigen Ab IgG >150 U/mL (positive >10.9), EBV Ab VCA IgG >600 U/mL (positive >21.9), and EBV Nuclear Antigen Ab IgG >600 U/mL (positive >21.9). Pediatric NPC differs from adult NPC due to its advanced locoregional disease and are most often undifferentiated with a strong association with EBV.

According to the World Health Organization, there are 3 different histologic subtypes of NPC. Keratinizing squamous cell carcinoma (WHO type 1) represents about 25% of all NPCs and has a weak association with EBV and an overall 5 year survival of 20–40%. Non-keratinizing differentiated carcinoma (WHO type 2) represents about 12% of all NPCs and has a strong association with EBV and an overall 5 year survival of 65%. Non-keratinizing undifferentiated carcinoma (WHO type 3) as seen in our patient is the most common subtype accounting for >60% of all NPCs and is the most frequent pediatric subtype. It has a strong association with EBV and a 5 year survival of 73–95% (4). Compared to adults, pediatric patients have a significantly greater overall and relapse-free survival (5, 6).

The gold standard for initial diagnosis of NPC is with nasopharyngeal endoscopy with biopsy and radiologic imaging to aid in the staging and extent of disease. CT and MRI are often performed with MRI being the preferred imaging modality (7, 8). T1 images typically show an asymmetric mass which is hypo to isointense to muscle and T2 images shows moderate hyperintensity. Invasion of the mass into the skull base is more readily detected on MRI due to changes of the marrow signal of the clivus (decreased on T1 and increased on T2) (9). Prior to the initiation of therapy, PET/CT is often used for initial staging to assess for local and distant metastasis as in our case which demonstrated a single metastatic left cervical node. Although the MRI report noted bilateral level 2 cervical lymph nodes, PET/CT was able to localize the increased metabolic activity to a single left-sided level 2 cervical node.

## Conclusion

NPC is a rare pediatric malignancy that often goes undiagnosed until a later stage. This case highlights the importance of recognizing the signs and symptoms of NPC tumors and the importance of radiographic imaging. As in our case, many of the pediatric cases of NPC tend to be diagnosed at a later stage due to the non-specific symptoms.

## Author Contributions

RaM wrote and edited the manuscript and captured images. FV and HG captured pathology slides and assisted with figures. RiM captured MRI images and edited the manuscript. MO assisted in writing and editing the manuscript.

### Conflict of Interest Statement

The authors declare that the research was conducted in the absence of any commercial or financial relationships that could be construed as a potential conflict of interest.
